# Epitranscriptomic profiling of N6-methyladenosine-related RNA methylation in rat cerebral cortex following traumatic brain injury

**DOI:** 10.1186/s13041-020-0554-0

**Published:** 2020-01-28

**Authors:** Jiangtao Yu, Yuxian Zhang, Haoli Ma, Rong Zeng, Ruining Liu, Pengcheng Wang, Xiaoqing Jin, Yan Zhao

**Affiliations:** 1 Emergency Center, Zhongnan Hospital of Wuhan University, Wuhan, 430071 China; 2 Department of Biological Repositories, Zhongnan Hospital of Wuhan University, Wuhan, 430071 China; 3 Hubei Clinical Research Center for Emergency and Resuscitation, Zhongnan Hospital of Wuhan University, Wuhan, 430071 China

**Keywords:** m6A methylation, Epigenetic modification, FTO, Traumatic brain injury, Rat cortex

## Abstract

**Background:**

N6-methyladenosine (m6A) is the most prevalent post-transcriptional modification of eukaryotic mRNA. It has been reported that there is a stimulus-dependent regulation of m6A in the mammalian central nervous system in response to sensory experience, learning, and injury. The mRNA m6A methylation pattern in rat cortex after traumatic brain injury (TBI) has not been investigated.

**Results:**

In this study, we conducted a genome-wide profiling of mRNA m6A methylation in rat cortex via methylated RNA immunoprecipitation sequencing (MeRIP-Seq). After TBI, the expressions of *METTL14* and *FTO* were significantly down-regulated in rat cerebral cortex. Using MeRIP-Seq, we identified a total of 2165 significantly changed peaks, of which 1062 were significantly up-regulated and 1103 peaks were significantly down-regulated. These m6A peaks were located across 1850 genes. The analysis of both m6A peaks and mRNA expression revealed that there were 175 mRNA significantly altered methylation and expression levels after TBI. Moreover, it was found that functional FTO is necessary to repair neurological damage caused by TBI but has no effect on the spatial learning and memory abilities of TBI rats by using FTO inhibitor FB23–2.

**Conclusion:**

This study explored the m6A methylation pattern of mRNA after TBI in rat cortex and identified FTO as possible intervention targets in the epigenetic modification of TBI.

## Background

More than 5.3 million people in the United States suffer from disability caused by traumatic brain injury (TBI) each year, and 1.7 million people sustain brain trauma, resulting in more than 60 billion in economic losses [1]. The causes of TBI include blast injury, sports injury, and penetration of the skull. TBI patients suffer from both short-term and long-term effects of their injuries. More than half of the severe TBI survivors are severely disabled within one year after injury, and some patients have devastating neurological disorders, including cognitive or memory impairment, and motor, sensory or emotional function obstacles [2]. In addition, for some patients with mild repetitive TBI, such as athletes in contact sports may develop chronic traumatic encephalopathy in later life [3]. The pathophysiological mechanism of TBI has two aspects, the primary injury caused by direct mechanical force and the secondary injury of neurons. The mechanical force of the impact directly impairs the axon and a part of a neuron. The mechanism involved in secondary injury is more complicated [3–5]. It includes delayed cell death (such as the newly discovered ferroptosis [6]), brain edema, metabolic defects [7], and blood-brain barrier damage caused by biochemical and physiological changes after trauma [8].

Recently, a series of studies have found that epigenetic changes play important roles in TBI-induced pathophysiological responses such as DNA methylation, chromatin post-translational modifications, and miRNA regulation of gene expression [5, 9, 10]. Epigenetics refers to the up- or down-regulation of gene or protein expression without changing the DNA sequence [11]. Bailey and colleagues demonstrated that exposure to simulated free-field explosions resulted in a significant increase in ten-eleven translocation methylcytosine dioxygenase in the prefrontal cortex and hippocampus of male rats [9]. The TET protein family has been proposed as a viable mechanism for active demethylation, and thus these changes may be associated with a higher degree of hypomethylation in the rat brain [12, 13]. *GADD45* has been identified as a mediator of demethylation of active DNA. In a combined spinal and neurological injury model, systemic folate therapy increases the methylation of the *GADD45a* promoter at 12 of its 18 CpG sites, restoring its methylation level to baseline and promoting spinal cord regeneration [14, 15]. TBI induced extensive changes in the DNA methylation patterns of rat hippocampus and the differentially methylated gene sites within 10 kb distance [16]. Another study investigated DNA methylation alterations after TBI in the rat frontal cortex using the brain blast-induced injury model and found that these differentially methylated genes were enrichet in cell death, survival, and nervous system development and function [17].

N6-methyladenosine (m6A) is the most prevalent post-transcriptional modification of eukaryotic mRNA and long non-coding RNA [18]. Adenosine in cellular RNA can be chemically modified by adding a methyl group at the N6 position of the adenine base, thereby producing a m6A adenosine, which is a nucleoside and a part of ribose (ribofuranose) and adenine. The composition is linked in the middle by a β-N9-glycosidic bond [19]. There are three basic mechanisms in m6A methylation: “writers” are methyltransferases including METTL3 and some related proteins like METTL14; “readers” are m6A binding proteins involved in the translation process; “erasers” are demethylases, including ALKBH5 and FTO [20, 21]. According to previous studies, m6A methylation was of great significance in the maintenance and differentiation of embryonic stem cells [22], the development and maintenance of acute myeloid leukemia [23], and the self-renewal of leukemia stem cells/initiating cells [24]. Bioinformatics analysis indicated that the neuron subtype-specific gene region was enriched for m6A. At the level of a single neuron, m6A-modified RNA and its interactions were spread over specific structures such as axons, dendrites, presynaptic nerve endings, and dendritic spines [25]. It has been demonstrated that there is a stimulus-dependent regulation of m6A in the mammalian central nervous system in response to sensory experience, learning, and injury [26, 27]. *METTL3* knockdown results in a prolonged cell cycle of cortical neural progenitor cells and decreased differentiation of radial glial cells. Knockout of *METTL14* in mouse embryos leads to prolongation of cortical neurogenesis to the postnatal stage. These data indicate a close relationship between m6A and nerve cells [28]. Damage to nerve cells in various regions of the adult nervous system can strongly induce regeneration. This recovery process relies on rapid de novo gene transcription, as well as protein synthesis. Using a peripheral sciatic nerve injury model, up-regulation of m6A in dorsal root ganglion neurons was reported to occur within three days after injury [29].

Compared with DNA modifications in TBI, the roles of RNA methylation have not been elucidated to date. In order to explore the epigenetic modifications of RNA in TBIand their functions in neurogenesis after TBI, we analyzed the N6-methyladenosine methylation profiles in the cortex of controlled cortical injury (CCI) rats. The CCI model in rats is a widely used TBI model [30]. In addition, we also analyzed the differential expressions of mRNA after TBI. Analysis of RNA and m6A methylation levels, we have identified potential novel targets that can provide a basis for further intervention in TBI.

## Methods

### Animals

Male Sprague-Dawley rats (aged 10 weeks, each weighting approximately 250–300 g) were purchased from Vital River Laboratory Animal Technology Co. Ltd. (Vital River, China). Each rat was housed in a regular environment of 12 h light/dark cycle with free access to water and food for one week in the Animal Experimental Center of Zhongnan Hospital of Wuhan University. The weight of each animal was controlled (300–350 g) during experiments. The procedures of this study were carried out in accordance with the guidelines of the Chinese Council on Animal Protection, and approved by the Institutional Animal Care and Use Committee of Wuhan University (IACUC: 2019010). We made every effort to reduce the suffering of animals in the experiments. The anesthesia method was intraperitoneal injection of 5% pentobarbital (50 mg/kg). In MeRIP-Seq experiments, 18 rats were randomly divided into 6 groups, and each group had 3 rats. Half of the groups were randomly assigned as TBI groups and the other half were sham operation groups (i.e., only cranial incision was performed). The MeRIP-Seq required at least 100 μg RNA in each sample and ~ 50 μg RNA in each rat cortex; therefore, the RNA of 3 rats were pooled as one sample for MeRIP-Seq and there were 3 samples for TBI and Sham groups, respectively.

### Animal model of controlled cortical injury

The controlled cortical impact (CCI) was chosen to establish the animal model of TBI as described previously [31, 32]. After intraperitoneal injection of pentobarbital, a 5 mm diameter hole was drilled over the right hemisphere bone equidistant between the lambda and bregma. Then the rats were put on the CCI device (Custom Design & Fabrication, USA). The 3 mm impactor tip was placed at the center of the craniotomy to induce moderate injury at 5 m/s speed, 200 msec dwell time, and 2 mm deformation depth. Any rats with a herniation of dura mater or occlusion of the injury hub were eliminated from the group. Rats were housed in separate cages after surgery and had free access to food and drink for 24 h. After 24 h, rats were fully anesthetized with 5% pentobarbital (50 mg/kg) and the undamaged tissues around the wound were surgically collected and were refrigerated at − 80 °C until RNA extraction.

### RNA extraction and quantitative reverse transcription polymerase chain reaction

The total RNA was extracted from the cortical tissues of sham and TBI groups. In quantitative reverse transcription polymerase chain reaction (qRT-PCR) experiments, these were 6 rats each in TBI and Sham groups. The frozen tissue samples were first crushed using a frozen mortar and were ground for two minutes until the samples became powder. Then each sample with 50–100 mg fresh weight was added to 1 mL of TRIzol (Thermo Fisher Scientific, USA) and mixed vigorously. After adding 200 μL of chloroform, shaking by hand for 15 s, and incubating at room temperature for 5 min, centrifugation was carried out at 13,000 rpm, 4 °C for 15 min. The aqueous phase was then transferred to a 1.5 mL RNase-free centrifuge tube. An equal volume of 100% isopropanol was added to the aqueous phase, and after incubating at room temperature for 10 min, it was centrifuged at 13,000 rpm for 15 min at 4 °C. Finally, the supernatant was removed. The precipitate was washed by adding 1 mL of cold 75% ethanol and centrifuged at 13,000 rpm for 5 min at 4 °C. The wash solution was discarded and the RNA pellet was air dried for 5–10 min. Finally, the RNA was dissolved in 50 μL RNase-free water.

To analyze the expression levels of genes encoding m6A methylation enzymes, reverse transcription polymerase chain reaction (qRT-PCR) was performed. A total of 1 μg of RNA was reverse transcribed into cDNA using the ReverTra Ace qPCR RT Kit (TOYOBO, Japan) following the manufacture’s instructions; the cDNA products were diluted 10-fold for further use. Then the PCR was performed with 2 μL diluted cDNA using the ReverTra Ace qPCR RT Kit (TOYOBO, Japan) in the PCR instrument (CFX Connect™, Bio-Rad, USA). *Actb* was used as the internal control to normalize the expression levels of candidate genes. The primers used in qRT-PCR were designed using the Primer3 website (http://primer3.ut.ee/) and listed in Additional file 1: Table S1.

### Methylated RNA immunoprecipitation sequencing (MeRIP-Seq)

The MeRIP-Seq method was used to measure the methylation levels of m6A in RNA. The N6-methyladenine antibodies were used to enrich highly methylated RNA fragments for high-throughput sequencing to detect m6A modifications at the entire transcriptome range as previously described Briefly, after adding 250 μL of fragmentation buffer (10 mM ZnCl_2_, 10 mM Tris-HCl pH 7.0) to the 50 μL of isolated 2 × poly-A RNA to a final volume of 300 μL, intact poly-A purified RNA was denatured at 95 °C for 5 min using a thermocycler. The fragmentation reaction was stopped by adding 50 μL of Stop Buffer (0.5 M EDTA) to a final volume of 350 μL and immediately put on ice. The fragmented RNA was allowed to bind with m6A-Dynabeads at room temperature while rotating at 7 rpm (tail flip) for 1 h. The m6A-Dynabeads-RNA complex was resuspended in 500 μL of m6A binding buffer and incubated for 3 min at room temperature. The supernatant was removed after placing the beads in the magnet. The next step was to add 125 μL of 42 °C pre-heated Elution Buffer to the m6A-Dynabead complexes and incubate at 42 °C for 5 min. Then, the m6A positive RNA was purified by adding 500 μL of acid phenol-chloroform to the sample collected in the previous step. We took 100 ng of RNA for each sample (100 ng of input and 100 ng of post m6A-IP positive fraction) for library construction utilizing the Stranded mRNA-seq Kit (Illumina, USA). After the sequencing library was established, all samples were sequenced by Illumina Hiseq X10 (OE Biotech, China).

### Behavioral testing: modified neurological severity score and Morris water maze

FTO inhibitor FB23–2 (MedChemExpress, USA) was used to block FTO demethylase function by intraperitoneal injection (1.4 mg/kg) 15 min after CCI [33]. The 25 rats were randomly divided into 4 groups: TBI + FB23–2 (*n* = 7), TBI + DMSO (*n* = 8), Sham+FB23–2 (*n* = 5), and Sham+DMSO (n = 5). A modified neurological severity score (mNSS) consisting of motor, sensory, balance, and reflex tests, was used to estimate the neurological deficits in rats [34]. The higher the mNSS score, the greater the neurological dysfunction. We use the Morris water maze (MWM) test to assess spatial learning ability [35]. The experimental training phase was carried out continuously for 5 days, with training 4 times a day. During training, the rats were placed into the pool from the four water entry points facing the pool wall, and the time required for the rats to enter the water to find the underwater hidden platform and stand on it was recorded as the incubation period. The rats were allowed to stand on the platform for 15 s after locating it. If the rat failed to find the platform within 60 s after entering the water, it was gently dragged onto the platform from the water, staying for 15 s before removal for the next training. At day 6 post CCI, we recorded the escape latency of each rat.

### Data processing and bioinformatics analyses

Raw data (raw reads) of fastq format were first processed using the Trimmomatic software [36]. Clean data (clean reads) were obtained by removing reads containing adapter, reads containing ploy-N, and low-quality reads from raw data. We then randomly extracted 250,000 paired reads from clean data, using the BLASTn software to align with the NT database (https://ftp.ncbi.nih.gov/blast/db) and take the best result with e-value <1e-10 and coverage > 80%. Meanwhile, the SortMeRNA software was used to discard ribosomal RNA (rRNA) reads and the remaining clean reads were mapped to the reference genome using HISAT2 with default parameters. Unique reads with high mapping quality were retained and potential PCR duplicates were removed using Picard [37, 38].

To access the quality of methylated RNA immunoprecipitation sequencing data, the Trumpet R package was used for evaluation of m6A-seq data quality [39]. The m6A-enriched peaks in each m6A immunoprecipitation sample were identified via MeTDiff peak calling software with the corresponding input sample serving as control. The MeTDiff was run with options (FRAGMENT_LENGTH = 200, PEAK_CUTOFF_PVALUE = 0.01, and PEAK_CUTOFF_FDR = 0.05) for peak detection [40]. Called peaks were annotated by intersection with gene architecture using ChIPseeker [41]. Differential analysis for m6A-Seq identified differences in RNA methylome in a case-control study and the differential peaks were detected by MeTDiff with parameter (FRAGMENT_LENGTH = 200, PEAK_CUTOFF_PVALUE = 0.01, DIFF_PEAK_CUTOFF_FDR = 0.05, and PEAK_CUTOFF_FDR = 0.05), then differential peaks were annotated by ChIPseeker.

GO enrichment and KEGG pathway enrichment analyses of peaks and differentially expressed peaks were performed respectively using R based on the hypergeometric distribution. The statistics in this study were analyzed using SPSS Version 25.0 software (IBM, USA). The network of m6A peak and mRNA expression were constructed using the Cytoscape Version 3.7.2 software.

## Results

### *METTL14* and *FTO* were both down-regulated after TBI in rat cortex

**Fig. 1:**
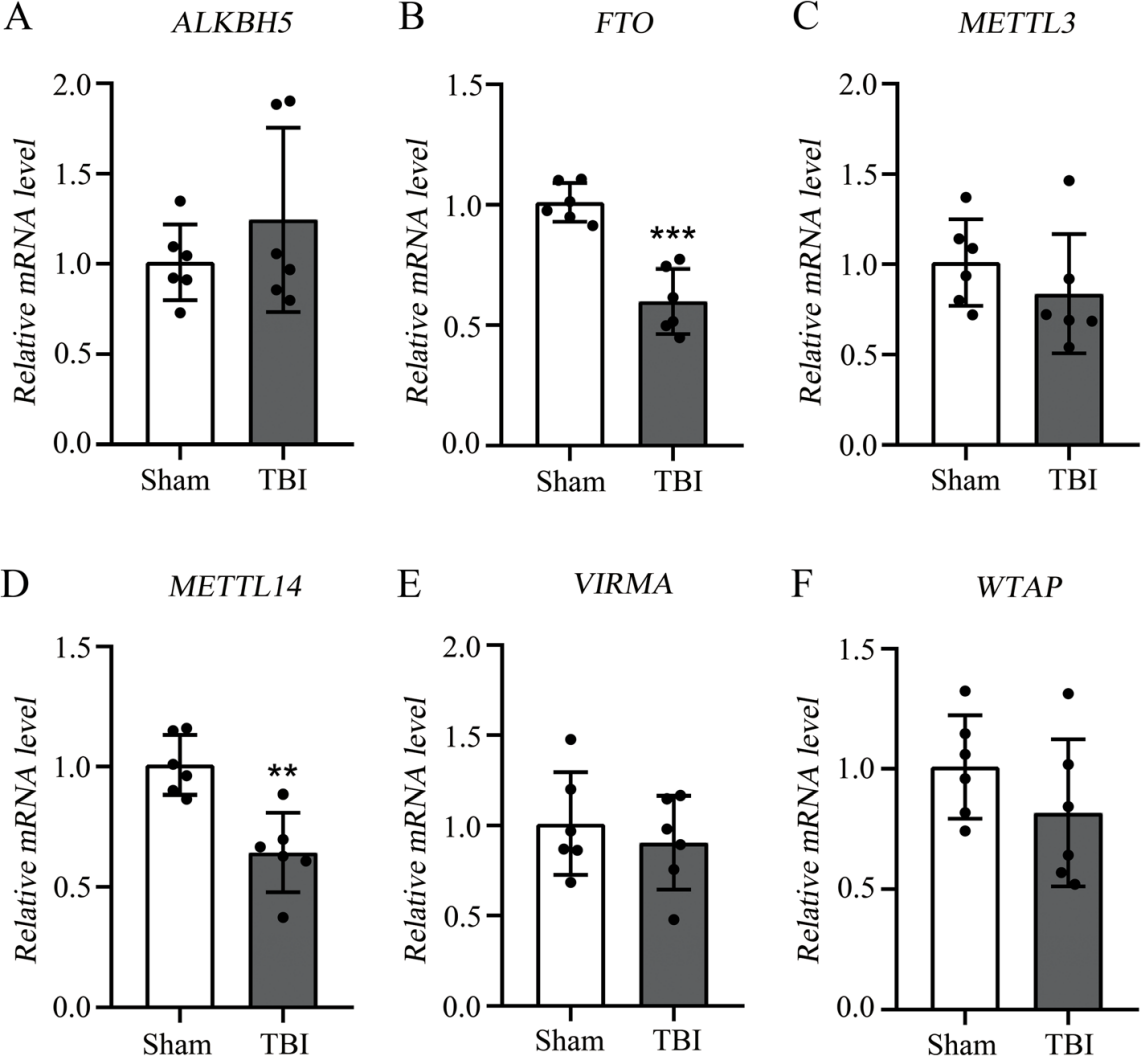
Reverse transcription polymerase chain reaction (RT-PCR) of *METTL3*, *METTL14*, *WTAP*, *VIRMA*, *FTO*, and *ALKBH5.* **a**–**b**, RT-PCR of demethylases *ALKBH5* and *FTO*, and the *FTO* is significantly down-regulated after TBI. **c**–**f**, RT-PCR of methyltransferases *METTL3*, *METTL14*, *VIRMA*, and *WTAP*, and the *METTL14* is significantly down-regulated after TBI. TBI group (*n* = 6); Sham group (*n* = 6). “**” and “***” indicate *p* < 0.01 and *p* < 0.001, respectively

In order to investigate whether m6A methylation status was changed in rat cortex after TBI, we evaluated the six genes encoding methyltransferases and demethylases by qRT-PCR: *METTL3*, *METTL14*, *WTAP*, *VIRMA*, *FTO*, and *ALKBH5*. The expression of *METTL14* (methylation enzyme) and *FTO* (demethylation enzyme) were both significantly down-regulated in the TBI group compared with the sham group (*p* < 0.05). The expression of the other four genes including *METTL3*, *WTAP*, *VIRMA*, and *ALKBH5* were not significantly changed in the rat cortex after TBI. The expression changes of *METTL14* and *FTO* could lead to a dynamic change in the m6A methylation in the rat cortex after TBI (Fig. 1).

### Overview of methylated RNA immunoprecipitation sequencing results

To measure the m6A methylation level of the sham and TBI groups, we performed a transcriptome-wide m6A-seq analysis using MeRIP-Seq. We found an average of 11.67 Gb sequencing data in the MeRIP-Seq samples and 13.46 Gb sequencing data of input samples were generated (Additional file 2: Table S2). After removing the adaptor sequence, low-quality reads, and low-quality bases from the 3′ and the 5′ terminals, the average number of clean data was 10.66 Gb in the MeRIP-Seq samples and 12.28 Gb in the input samples (Additional file 2: Table S2). The clean reads of each sample were compared with the rat genome to obtain positional information on the reference genome and an average of 95.43% reads was mapped on the rat genome. The clean reads with multiple alignments to the rat genome were eliminated (5.48%), and the clean reads with unique alignment retained for subsequent analysis (89.95%) (Additional file 3: Table S3).

### Topological distribution of m6A peaks

**Fig. 2:**
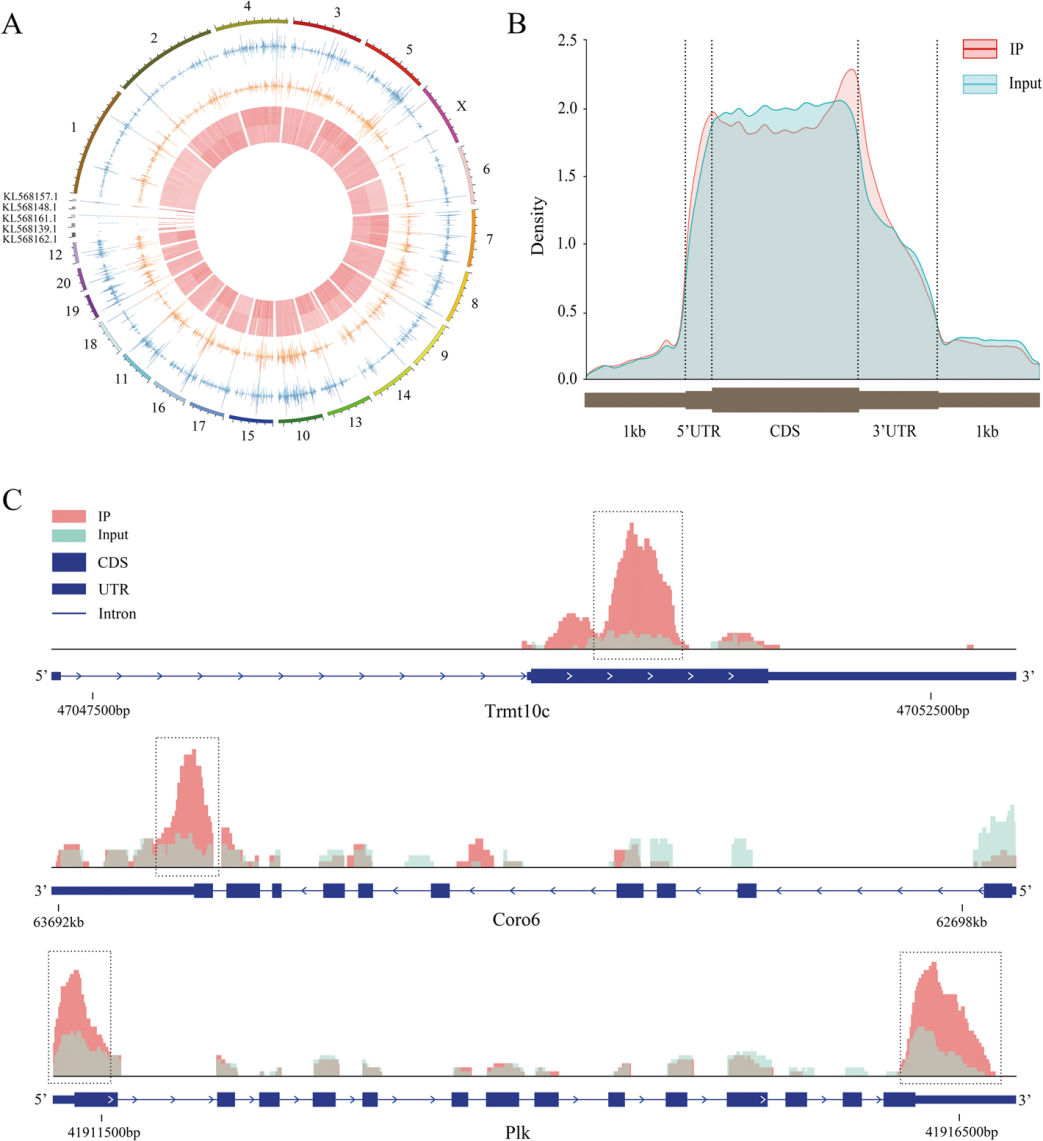
Topological distribution of m6A peaks. **a**, The distribution pattern of m6A peaks in different chromosomes. **b**, The distribution patterns of m6A methylation peaks in gene structures of mRNA. The peaks were mostly distributed on the exon region and there was a distinct enrichment peak at the 3′ terminate (near the stop codon) and an enrichment peak at the 5′ terminate (near the start codon). **c**, IGV plot shows directly the peaks in the genes of *Trmt10c*, *Coro6* and *Plk*. The peak of *Trmt10c* was located at the CDS region, the peak of *Coro7* was located at the CDS and 3′ UTR regions, and the peak of *Plk* was located at the 3′ UTR, the CDS, and the 5′ UTR regions

Compared with the sequencing data between MeRIP-Seq samples and their corresponding inputs, m6A peaks were identified, including 11,789 distinct m6A peaks for 4900 transcribed genes from all samples (Additional file 4: Table S4). The chromosomes with the most m6A methylation sites were chromosomes 1, 3, and 10 with 1386, 921, and 905 m6A methylation sites, respectively (Fig. 2a and Additional file 4: Table S4). Specifically, the number of m6A modification sites on the gene ranged from 1 to 19, with 64.31% of genes having one or two methylation sites and 35.69% of genes having 3 or more methylation sites (Additional file 4: Table S4). For instance, *Pclo* located on chromosome 4 had the maximum number of methylation sites (i.e., 19 sites) (Additional file 4: Table S4). In addition, we analyzed the distribution patterns of m6A methylation peaks in gene structures of mRNA and we found that the peaks were mostly distributed on the exon region, and there was a distinct enrichment peak at the 3′ terminate (near the stop codon) and a enrichment peak at the 5′ terminate (near the start codon). The reads in the input samples were higher than the reads in the IP samples at the CDS region (Fig. 2b). We have chosen three genes to show the m6A methylation pattern. The peak of *Trmt10c* was located at the CDS region, the peak of *Coro7* was located at the CDS and 3' UTR regions, and the peak of *Plk* was located at the 3' UTR, the CDS, and the 5' UTR regions (Fig. 2c).

### Significant m6A methylation changes after TBI

**Fig. 3:**
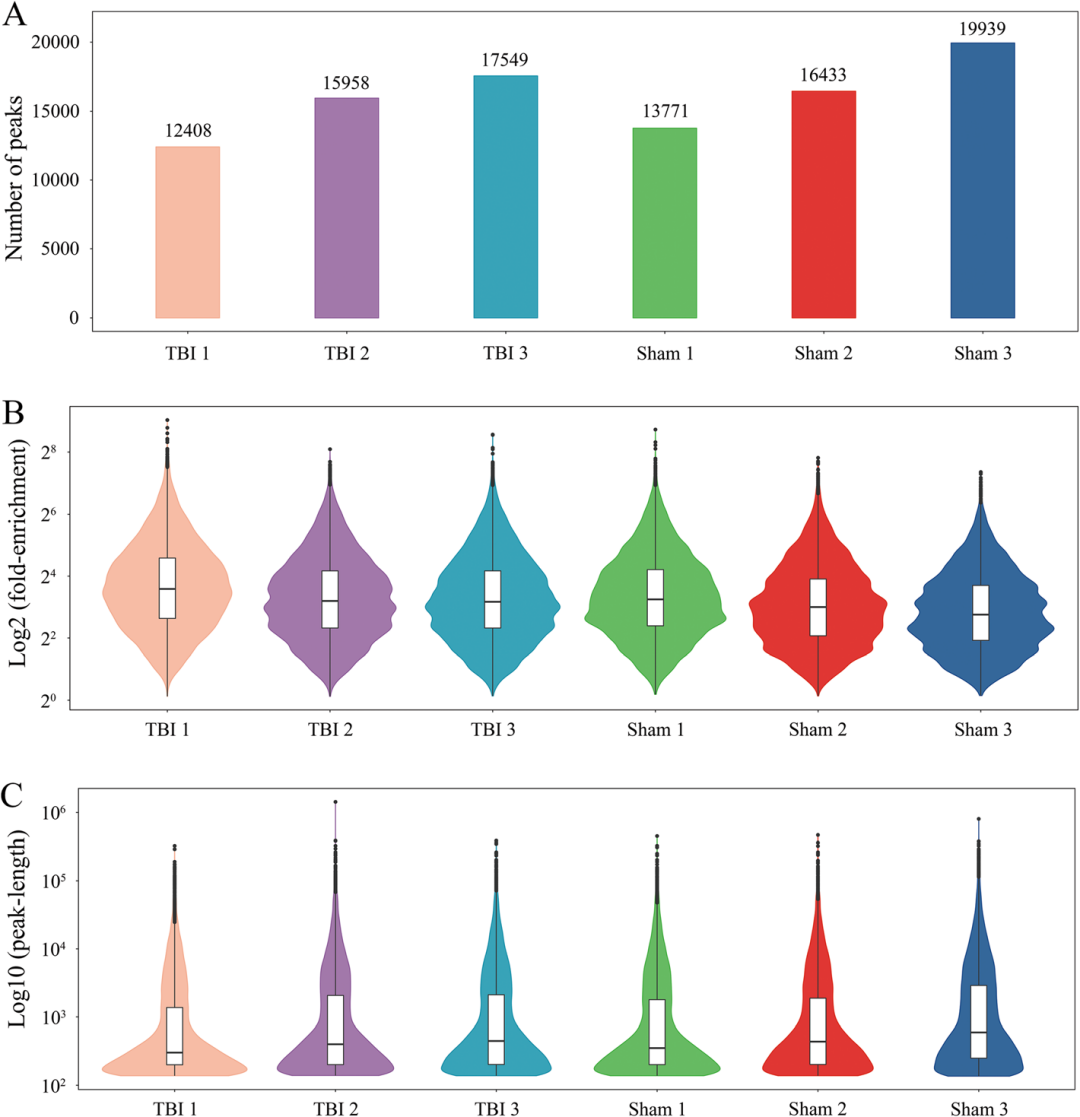
Number of peaks, fold enrichment of peaks and length of peaks in each group after TBI. **a**, The peak number of each group. An average of 15,305 peaks in the TBI group and an average of 16,714 peaks in the sham group were identified. **b**, The enrichment of each group. The average logarithmic fold-enrichment of TBI group was 4.11, and the average logarithmic fold-enrichment of sham group was 3.72. **c**, The peak length of each group. The average peak length of TBI group was 3426.14 bp, and the average peak length of sham group was 3831.73 bp

**Fig. 4:**
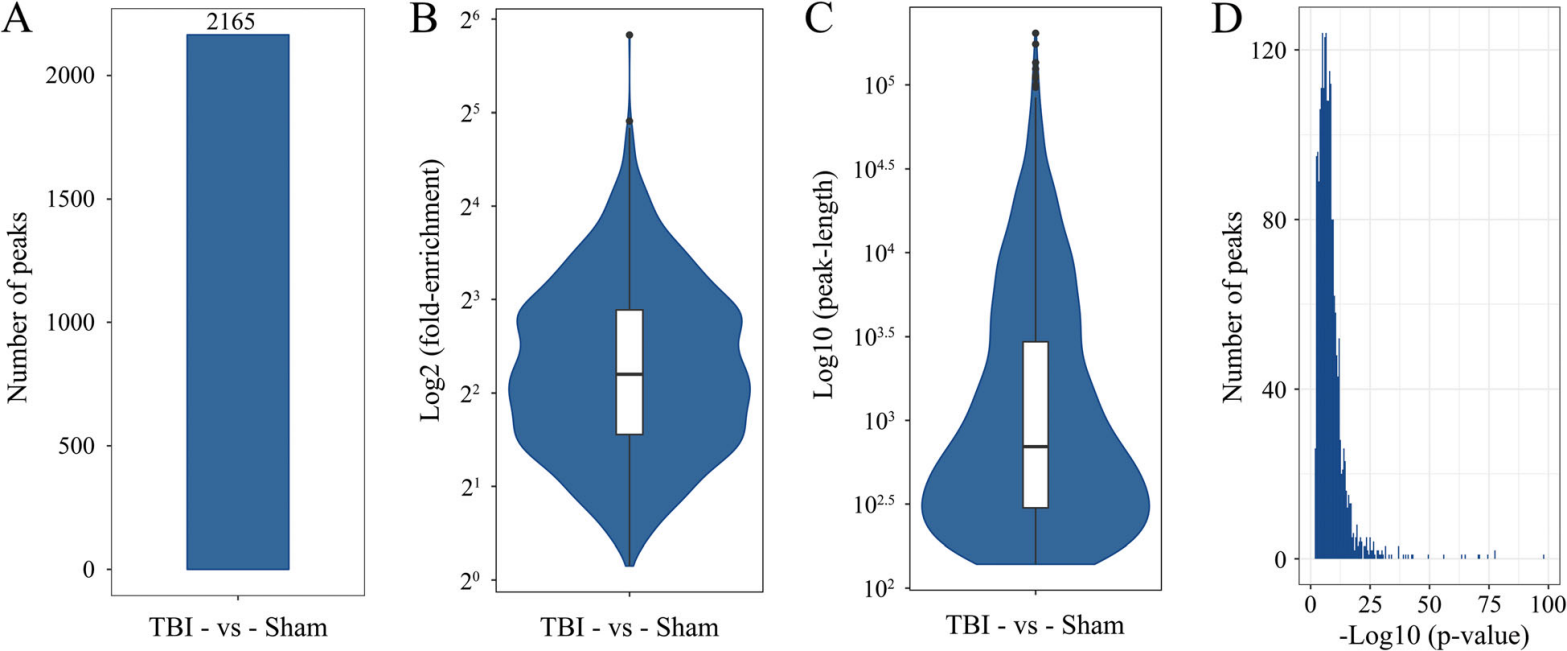
Peak number, fold enrichment, peak length and *p*-value in the TBI and Sham groups. **a**, A total of 2165 significantly changed peaks were identified (*p* < 0.01, FDR < 0.05). **b**, The average logarithmic fold-enrichment of the peaks were 2.53. **c**, The average length of each peak was 3.64 (log_10_). D, The distribution of *p*-value of all peaks

**Fig. 5:**
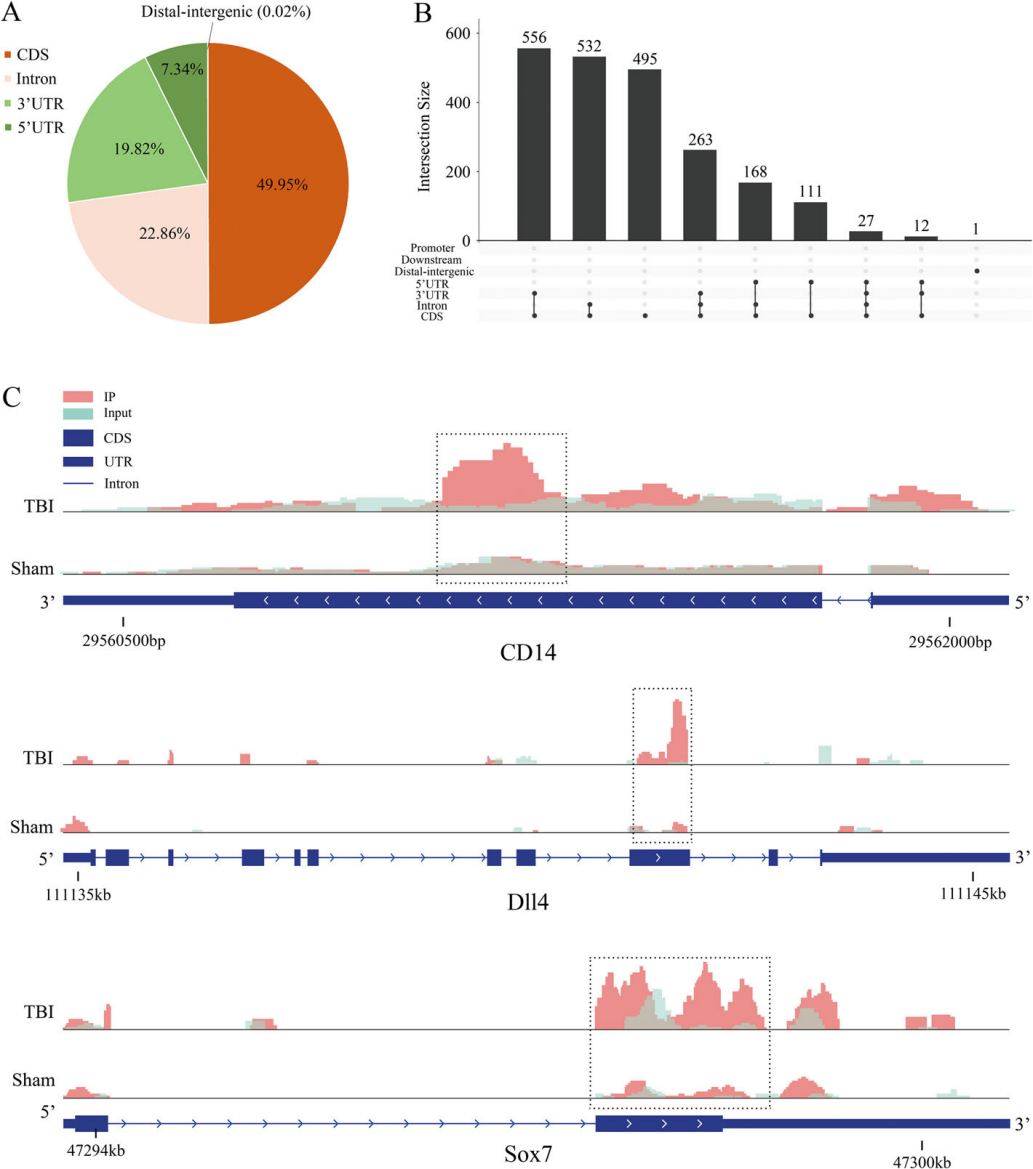
The distribution of significantly changed peaks after TBI. **a**, Sector graph shows the ratio of peaks in each region. The peaks with significant changes were mainly distributed in the exon (49.95%), CDS (22.86%), 3′ UTR (19.82%), and 5′ UTR (7.34%) and only 0.02% were found in the intergenic region. **b**, Showing the exact distribution pattern of significantly changed peaks post-TBI. There were 556 peaks distributed in the CDS and 3′ UTR regions, 532 peaks in the CDS and intron regions, and 495 peaks in the CDS region. **c**, Three representative genes with significantly changed peaks. The m6A level of CD14, Dll4, and Sox7 were significantly up-regulated after TBI by 3.63 fold, 2.10 fold, 3.14 fold, respectively

To reveal the roles of m6A methylation in TBI, we compared the methylation levels of rat cortex among the TBI group and sham group (Additional file 5: Table S5). An average of 15,305 peaks in the TBI group and an average of 16,714 peaks in the sham group were identified (Fig. 3a). Compared with the input sample, the average logarithmic fold-enrichment of TBI group was 4.11, and the average logarithmic fold-enrichment of sham group was 3.72 (Fig. 3b). The average peak length of TBI group was 3426.14 bp, and the average peak length of sham group was 3831.73 bp (Fig. 3c). A total of 2165 significantly changed peaks were identified (*p* < 0.01, FDR < 0.05), in which 1062 peaks were significantly up-regulated and 1103 peaks were significantly down-regulated (Fig. 4a). The top 20 differentially methylated mRNA were shown in Table 1. As illustrated above, 1580 out of 4900 genes had peaks that were differentially methylated. The average logarithmic fold-enrichment of all the peaks are 2.53 (Fig. 4b) and the average length of each peak was 4358.7 bp (Fig. 4c). The distribution of *p*-value among all peaks is shown in Fig. 4d. The peaks with significant changes are mainly distributed in the CDS (49.95%), intron (22.86%), 3′ UTR (19.82%), and 5′ UTR (7.34%), and only 0.02% were found in the intergenic region (Fig. 5a). Many peaks with significant changes spanned multiple genes structures; 556 peaks distributed in the CDS and 3′ UTR regions, 532 peaks in the CDS and intron regions, and 495 peaks in the CDS region (Fig. 5b). Three representative genes with significantly changed peaks are presented in Fig. 5c. The m6A level of *CD14*, *Dll4*, and *Sox7* were significantly up-regulated after TBI by 3.63 fold, 2.10 fold, and 3.14 fold, respectively.

**Table 1:** The top 20 significantly changed m6A methylation peak based on *p*-value

mRNA	Chromosome	Peak region	Peak start	Peak end	Lg (*p*-value)	Log_2_ (fold-change)	Up/Down
*Sp4*	6	3′ UTR	146,193,411	146,193,560	−434	−1.90	Down
*Mycbp2*	15	Exon	87,493,207	87,494,725	−434	−3.83	Down
*Pias4*	7	Exon	11,414,644	11,423,227	−8.36	−2.14	Down
*Fbxo32*	7	3′ UTR	98,065,124	98,067,070	−8.13	−1.43	Down
*Crhbp*	2	5′ UTR	25,152,370	25,153,284	−7.28	−1.61	Down
*Kcnj11*	1	3′ UTR	102,103,938	102,104,138	−7.15	−1.83	Down
*Amer3*	9	Exon	41,096,883	41,097,424	−7.00	−1.82	Down
*Spg7*	19	3′ UTR	55,913,654	55,913,954	−6.85	−2.99	Down
*Slc2a13*	7	5′ UTR	132,757,358	132,757,508	−6.62	−2.47	Down
*Trpc6*	8	Exon	6,872,050	6,872,448	−6.62	−1.50	Down
*Slfn4*	10	5′ UTR	70,428,465	70,428,814	−7.21	1.81	Up
*Mdfic*	4	3′ UTR	42,280,721	42,280,870	−6.79	4.17	Up
*Prss35*	8	3′ UTR	94,438,220	94,439,065	−6.27	1.05	Up
*Ppp1r3b*	16	3′ UTR	60,418,476	60,418,677	−6.18	3.14	Up
*Nlgn3*	X	Exon	71,221,689	71,222,484	−6.18	1.03	Up
*Hcn1*	2	Exon	50,495,344	50,499,750	−6.13	1.12	Up
*Rps6kb1*	10	5′ UTR	73,854,215	73,865,364	−6.08	1.49	Up
*Gpkow*	X	3′ UTR	15,638,954	15,640,719	−6.02	2.39	Up
*Prelid3a*	18	3′ UTR	63,213,663	63,215,734	−5.87	2.87	Up
*Pprc1*	1	Exon	265,818,641	265,818,890	−5.80	4.01	Up

### GO and KEGG analyses revealed the biological information behind m6A methylation

**Fig. 6:**
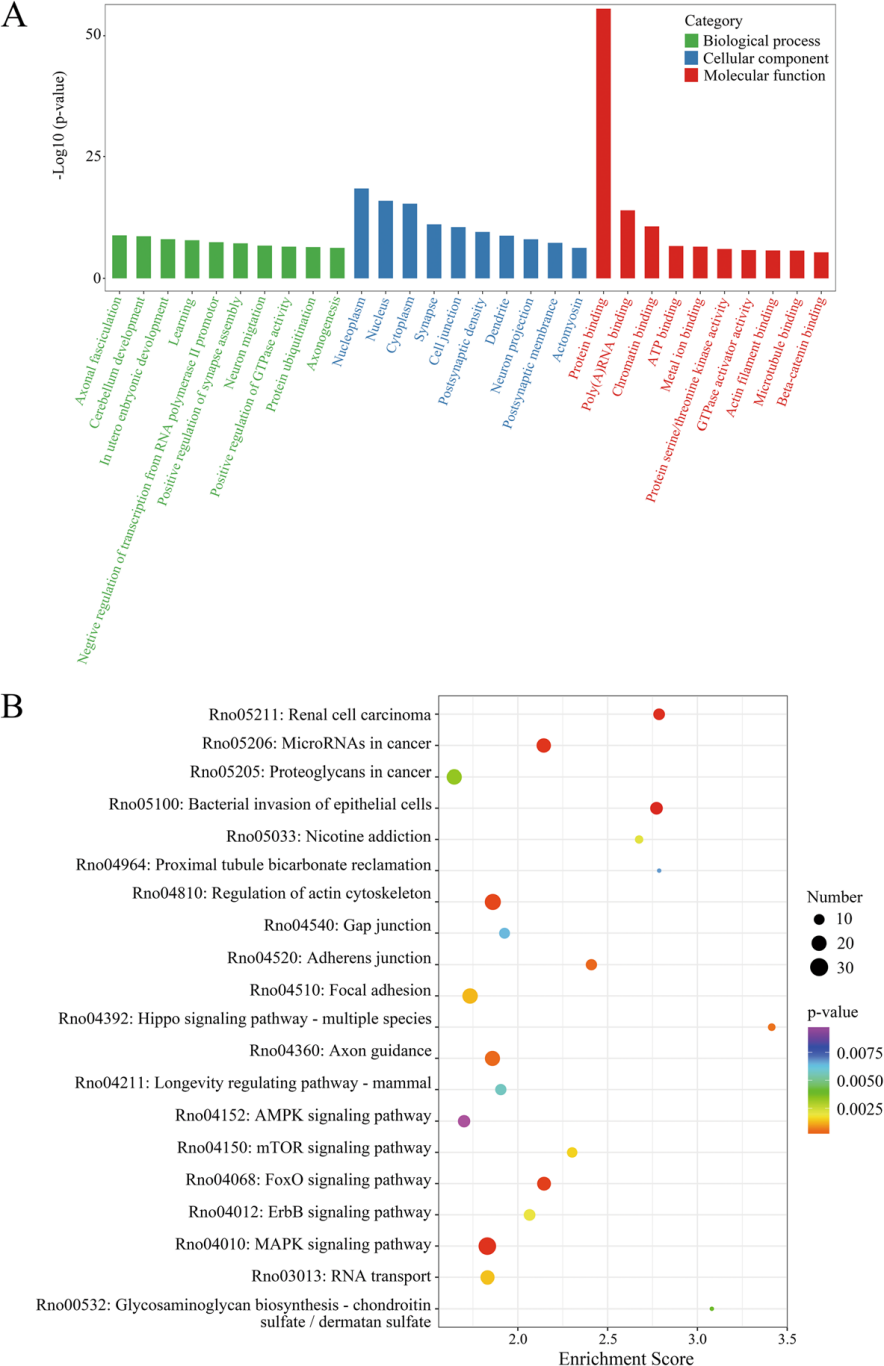
GO and KEGG analyses revealed the biological information behind mRNA m6A methylation. **a**, The top 10 enriched GO terms of the m6A peaks. **b**, The top 20 enriched pathways of m6A peaks

To clarify the function of RNA m6A methylation in TBI, we subjected the genes with significantly changed m6A methylation level to gene functional enrichment analysis using the Gene Ontology (GO) and Kyoto Encyclopedia of Genes and Genomes (KEGG). GO aims to establish a unified paradigm to explain the role of eukaryotic genes and proteins in cells. GO analyses showed m6A methylation was related to three parts of biological information. Molecular function: protein binding, poly(A) RNA binding, chromatin binding; cellular component: nucleoplasm, nucleus, cytoplasm; biological process: axonal fasciculation, cerebellum development, in utero embryonic development (Fig. 6a). Through KEGG analyses, we annotated the genes with significantly changed m6A methylation levels. These genes were mostly enriched in the pathway including renal cell carcinoma, microRNAs in cancer, and proteoglycans in cancer (Fig. 6b).

### Gene expression profiles after TBI

**Fig. 7:**
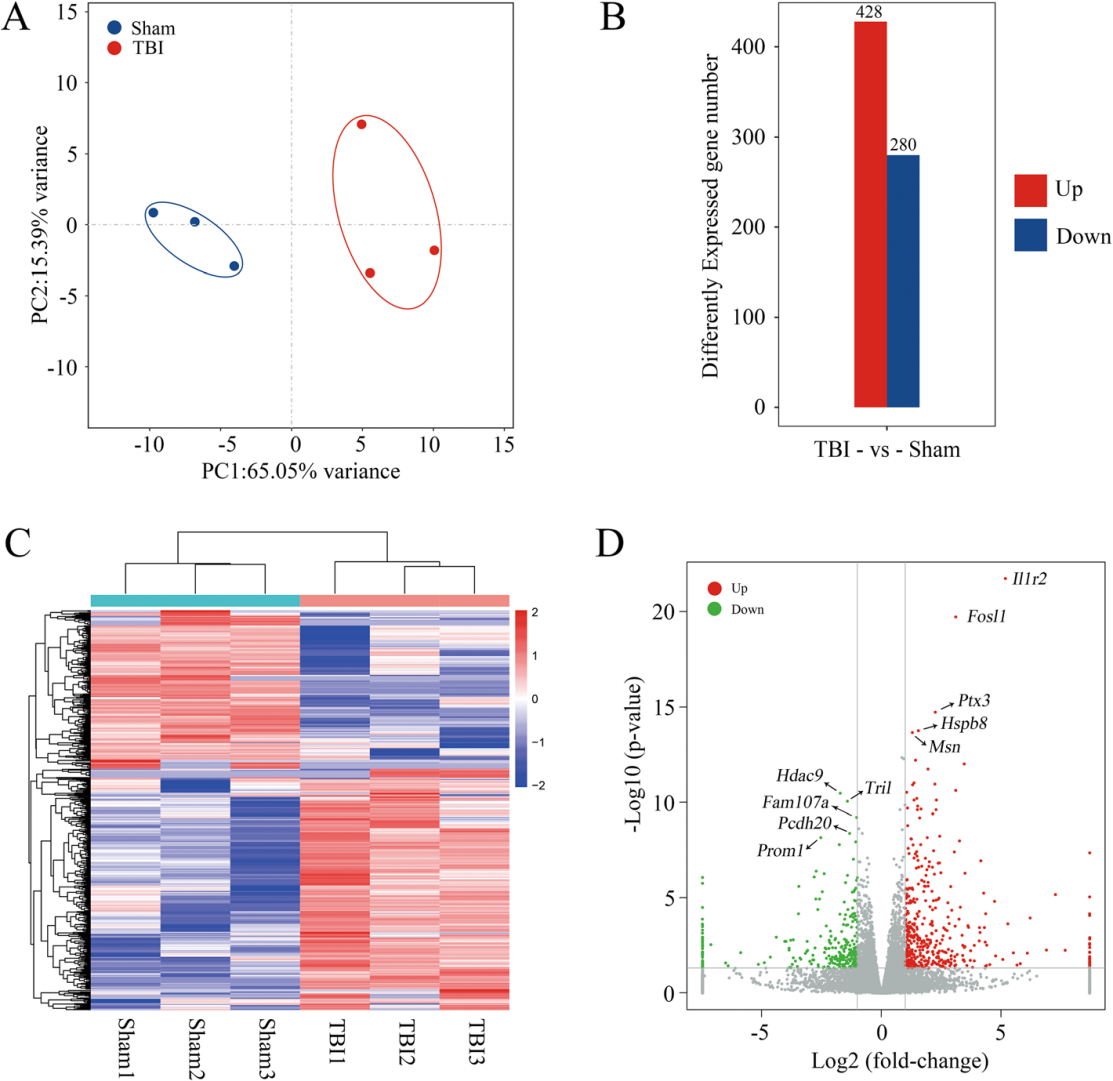
mRNA changes after traumatic brain injury. **a**, Principal component analysis of the TBI group and the sham group. The confidence ellipses of samples among sham and TBI groups were separate from each other. **b**, The expression level of mRNA post-TBI. A total of 428 mRNA expression increased and 280 mRNA expression decreased (*p* < 0.05, log_2_FC > 1) in rat cerebral cortex after TBI. **c**, Heat map shows the relative expression level of the three TBI IP and the three sham IP. **d**, Volcano plot shows the significantly up-regulated and down-regulated mRNA after TBI

In order to further clarify the relationship between m6A methylation level and gene expression, we measured the level of mRNA changes in rat cerebral cortex after TBI using the RNA sequencing data of input experiments (Additional file 6: Table S6). Figure 7a shows the principal component analysis of the TBI and sham groups. It was found that the confidence ellipses of samples among sham and TBI groups were separated from each other, indicating that the gene expression patterns were similar within the same treatment group, but significantly different between sham and TBI groups (Fig. 7a). It was found that a total of 428 mRNAs increased and 280 mRNAs decreased expression (*p* < 0.05, log_2_FC > 1) in rat cerebral cortex after TBI (Fig. 7b). The top 20 differentially changed mRNA are shown in Table 2. The heatmap shows the relative expression level of the three TBI IPs and the three sham samples in the same group have similar presentation (Fig. 7c). The volcano plot (Fig. 7d) shows the significantly up-regulated and down-regulated mRNA after TBI. We marked the top 5 most significantly up-regulated genes (*Il1r2*, *Fosl1*, *Ptx3*, *Hsbp8*, and *Msn*) and top 5 down-regulated genes (*Hdac9*, *Tril*, *Fam107a*, *Pcdh20*, and *Prom1*) in the volcano plot.

**Table 2:** The top 20 differently expressed mRNA based on *p*-value

mRNA	Chromosome	Gene start (bp)	Gene end (bp)	*p*-value	Log_2_ (fold-change)	Up/Down
*Il1r2*	9	46,840,992	46,881,264	1.86E-22	5.18	Up
*Fosl1*	1	220,826,560	220,835,066	1.92E-20	3.11	Up
*Ptx3*	2	158,097,843	158,103,653	1.91E-15	2.25	Up
*Hspb8*	12	45,905,371	45,920,013	1.77E-14	1.54	Up
*Msn*	X	65,226,748	65,295,810	2.22E-14	1.30	Up
*Cebpd*	11	89,008,008	89,009,146	6.16E-13	1.43	Up
*Lif*	14	84,482,674	84,500,642	9.96E-13	3.46	Up
*Emp1*	4	169,147,243	169,181,966	1.82E-12	1.95	Up
*Csf2rb*	7	119,554,354	119,568,776	9.85E-12	1.37	Up
*Cd44*	3	92,696,313	92,783,658	1.13E-11	2.24	Up
*Hdac9*	6	53,488,883	54,059,119	3.45E-11	−1.72	Down
*Tril*	4	83,967,696	83,972,540	9.06E-11	−1.42	Down
*Fam107a*	15	18,399,515	18,415,965	6.17E-10	−1.03	Down
*Pcdh20*	15	71,772,777	71,779,033	4.29E-09	−1.33	Down
*Prom1*	14	71,533,063	71,637,417	7.08E-09	−2.53	Down
*Slc38a3*	8	116,406,241	116,422,366	1.19E-08	−1.07	Down
*Itm2a*	X	78,490,866	78,496,847	1.66E-08	−1.76	Down
*RGD1559896*	19	57,396,606	57,422,093	9.56E-08	−1.17	Down
*Clic6*	11	32,655,616	32,699,382	4.06E-07	−2.72	Down
*Nat8f3*	4	117,490,035	117,490,721	5.63E-07	−2.39	Down

### Combined analysis of m6A methylation and gene expression after TBI

**Fig. 8:**
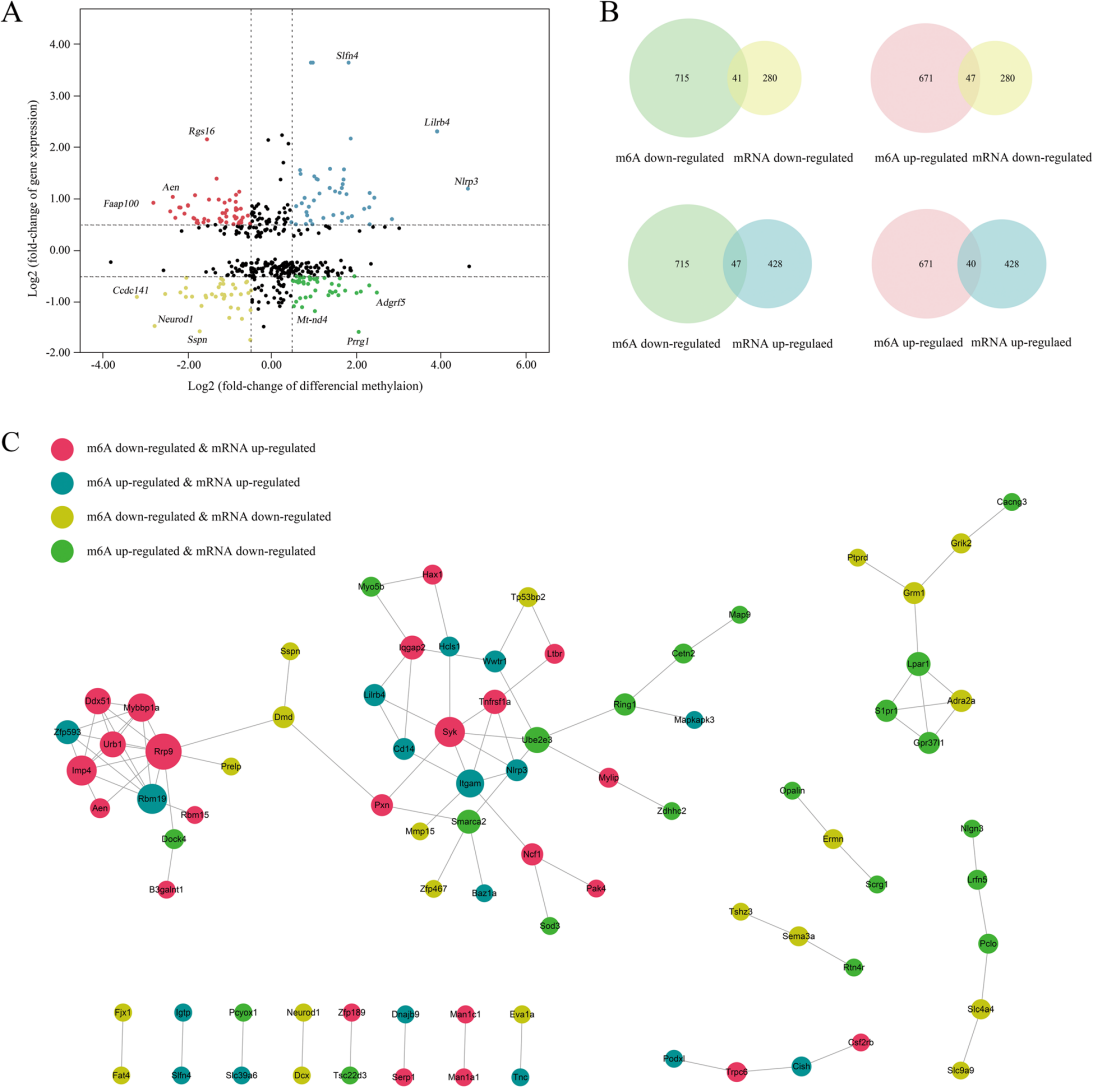
Joint analysis of m6A methylation and mRNA expression after TBI. **a**, **b**, Four quadrant graph and venn diagram shows the relationship between mRNA m6A methylation and its mRNA expression. **c**, The protein-protein interaction network shows the connection between the proteins of the genes used in the combined analysis

Combined analysis of m6A methylation and RNA expression levels used peaks with log_2_ foldchange > 0.5, *p* < 0.01 and the mRNA with log_2_ foldchange > 0.5, *p* < 0.05. As a result, there were 175 mRNAs where their m6A peaks and mRNA levels both changed significantly, among which the levels of 40 mRNAs were both up-regulated and the levels of 41 mRNAs were both down-regulated. Moreover, there were 47 genes with up-regulated mRNA expression and down-regulated m6A peaks and 47 genes with down-regulated mRNA expression and up-regulated m6A peaks (Additional file 7: Table S7). The relationship between m6A methylation and mRNA expression are shown in the quadrant graph and Venn diagram (Fig. 8a and b). Last, we conducted the protein-protein interaction network to show the connection between the proteins encoded by the 175 genes (Fig. 8c).

### FTO inhibitor FB23–2 increases the mNSS score of rats after TBI

**Fig. 9:**
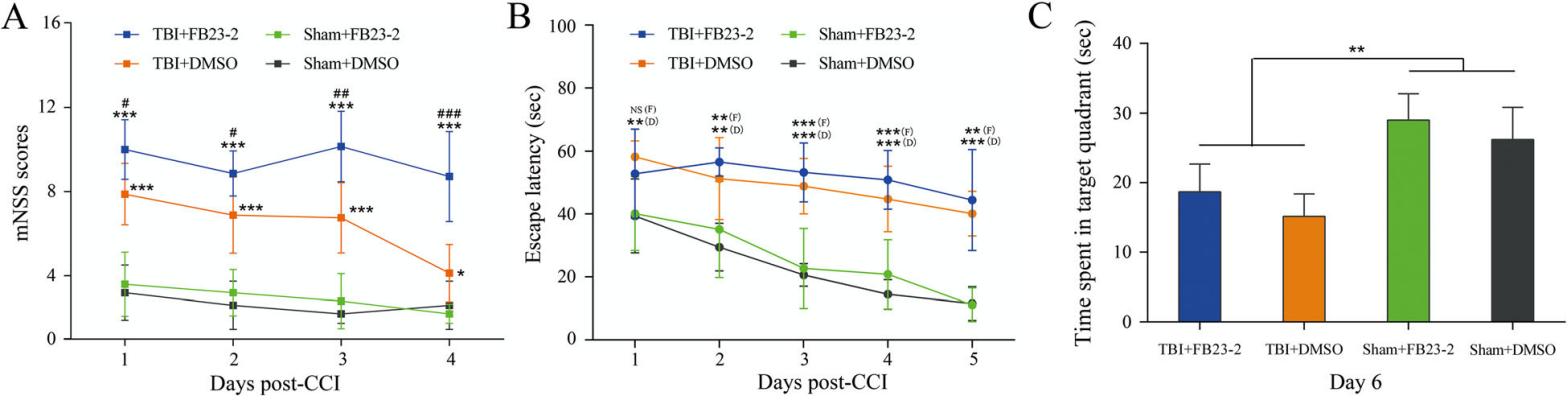
Effects of FTO inhibitior FB23–2 on neurological functions and cognitive and learning abilities in TBI. **a**, the score of four groups in mNSS: TBI + FB23–2 (*n* = 7), TBI + DMSO (*n* = 8), Sham+FB23–2 (*n* = 5), and Sham+DMSO (n = 5). **b** and **c**, the escape latency time and target quadrant time of four groups in MWM, respectively: TBI + FB23–2 (*n* = 7), TBI + DMSO (*n* = 8), Sham+FB23–2 (n = 5), and Sham+DMSO (n = 5). “*”, “**”, and “***” indicate *p* < 0.05, *p* < 0.01, and *p* < 0.001 in TBI vs Sham, respectively; “#”, “##”, and “###” indicate *p* < 0.05, *p* < 0.01, and *p* < 0.001 in FB23–2 vs DMSO. Error bars represent SD. NS indicates *p* > 0.05

In order to investigate the roles of m6A demethylase FTO in controlling rat neurological function after TBI, we performed the mNSS and MWM tests in the four groups: TBI + FB23–2, TBI + DMSO, Sham+FB23–2, and Sham+DMSO (Additional file 8: Tables S8 and Additional file 9: Table S9). The mNSS scores of sham+FB23–2 and the sham+DMSO group for four consecutive days showed no difference, indicating that FB23–2 has no effect on neurological function in normal rats. All TBI groups (i.e., TBI + FB23–2 and TBI + DMSO) scored significantly higher on the mNSS than the sham groups (i.e., Sham+FB23–2 and Sham+DMSO) for four consecutive days, indicating that CCI caused damage to neurological function. Interestingly, inhibition of FTO exacerbated damage to neurological function caused by TBI. Specifically, in the first day post-CCI, the mean score of TBI + FB23–2 group was 10, which was significantly higher than the TBI + DMSO group (7.875; *p* = 0.0135); On the second day post-CCI, the mean score of the TBI + FB23–2 group was 8.857, which was significantly higher than the TBI + DMSO group (6.875, *p* = 0.025). The trend continued on the third (*p* = 0.0018) and fourth (*p* = 0.0002) day post-CCI with the TBI + FB23–2 group scoring 10.14 and 8.71, while the TBI + DMSO group scored 6.75 and 4.13, respectively (Fig. 9a). However, the results of MWM test indicated that the spatial learning and memory abilities of TBI + FB23–2 and TBI + DMSO group showed no difference, indicating that inhibition of FTO don’t affect the spatial learning and memory of CCI rats (Fig. 9b and c).

## Discussion

An increasing amount of research has focused on the role of RNA m6A methylation in neurobiology and neurological diseases [24]. The transcriptome-wide profiling of RNA m6A in the prefrontal cortex of mice that received behavioral training revealed that RNA m6A methylation may be associated with memory consolidation [27]. It has been found that heterogeneity of RNA m6A methylation exist in the cerebellum and cortex of mice (methylation level and methylation site) through m6A-immunoprecipitation [29]. However, the RNA m6A methylation in rat cortex was not reported. Using MeRIP-Seq, we obtained a panorama of m6A methylation in rat cortex after TBI, which added more information to the role of m6A methylation in TBI epigenetics.

Compared with the sham group, the TBI group showed lower *METTL14* and *FTO* expression levels (Fig. 1). As one of the methyltransferases, *METTL14* can form a complex with *METTL3* to catalyze m6A methylation on RNA [19]. On the contrary, *FTO*, which had been considered a demethylase, could mediate m6A demethylation of RNA [42]. As a result of *METTL14* and *FTO* down-regulation in TBI, m6A methylation resulted in both increased peaks (1062) and decreased peaks (1103) after TBI (Fig. 4a). Previous studies focused on the methylation level of m6A in mouse hippocampus after brain injury found that the expression of *METTL3* was decreased but the expression of other enzymes (*METTL14*, *FTO*, *WTAP*, and *ALKBH5*) were not significantly changed [43]. The differences of m6A-related methyltransferases and demethylases between mouse hippocampus and rat cerebral cortex might be caused by interspecies differences or different organ sources. The differently methylated m6A peaks in TBI were mostly distributed at the 3′ UTR near stop codons and the 5′ UTR near start codons, which was consist with previous studies in both human and mouse [44–46]. Specifically, m6A distribution was highly enriched for 3’UTR (> 40%) mRNA, especially at the beginning of the last exon (within 150–400 NT), where it rose sharply (6-fold) [47].

The m6A methylation levels of several genes related to pathophysiological processes of TBI were significantly changed. The expression of *CD14* was an important part of the response by the central nervous system in acute inflammation after trauma [48]. There were more CD14^+^ cells in vascular peripheral and brain parenchyma after TBI within 1–2 days. *Dll4* was a protein- coding gene involved in the Notch signaling pathway as Notch ligand activates Notch1 and Notch4 [49, 50]. During spinal cord neurogenesis, *Dll4* inhibits V2a interneuron fate [51]. Recent studies have shown that *Dll4* and Notch signaling are involved in sprouting angiogenesis and artery formation, and the over expression of *Dll4* inhibits the vascular response [52, 53]. Interestingly, the expression level of *Dll4* and the m6A methylation level of *Dll4* were both up-regulated, which made the function of *Dll4* in TBI become unpredictable. *Bcl2* is part of the intrinsic apoptotic pathway and ceramide signaling pathway (affecting growth, proliferation, differentiation, apoptosis, and injury). The m6A peak of *Bcl2* was down-regulated after TBI, and the mRNA was also down-regulated.

A previous study suggested that m6A modification induced mRNA instability [54]. In order to better understand the roles of m6A methylation in TBI, we screened all the differently expressed peaks combined with the differently expressed mRNA. As a result, we identified 175 mRNA whose m6A peak and mRNA level both changed significantly, which could be divided into four outcomes: mRNA and m6A peaks both up-regulated (40), mRNA and m6A peak both down-regulated (41), the m6A peak up-regulated and mRNA peak down-regulated (47), and the m6A peak down-regulated and mRNA peak up-regulated (47). In our results, we didn’t observe a significant correlation between m6A methylation and mRNA expression. Previous research showed that m6A can both promote and inhibit of translation [55]. YTHDF2-mediated degradation controls the lifetime of target transcripts, while YTHDF1-mediated translation promotes translation efficiency. Thus the exact protein level of these m6A methylated mRNA could not be foretold, which remains to be further researched.

To verify the roles of m6A demethylase FTO in TBI rats, we inhibited the demethylation functions of FTO by using FTO inhibitor FB23–2, and then performed behavioral experiments. FB23–2 can inhibit the m6A demethylase FTO, and it has been confirmed in leukemia studies that the degree of m6A methylation is reduced after intraperitoneal injection of FTO [33]. In the mNSS experiments, the neurological function of rats after TBI presents a tendency to restore itself, which is in agreement with previous studies [56]. However, the self-healing phenomenon was not found in FB23–2-injected TBI rats, indicating that functional FTO is necessary to repair neurological damage caused by TBI. Corroborating this, we detected FTO mRNA in rat cerebral cortex after TBI by qRT-PCR. Taken together, we believe that FTO plays an important role in the maintenance of neurological function in TBI rats. Besides, the escape latency and the time spent in target quadrant post-CCI of TBI + FB23–2 group and TBI + DMSO group showed no difference in MWM test, indicating that FB23–2 has no effect on the spatial learning and memory abilities of TBI rats. Based on above results, the inhibition of FTO result in the exacerbate the neurological damage, and this result may imply that the increase of FTO expression level and enhancement of FTO function could be a solution to accelerate neurological repair after TBI. And the We suggested that adenovirus transfection of construct that increased FTO expression to further verify the role of FTO in TBI. This study also found that METTL14 is reduced after TBI, but unfortunately there is currently no effective METTL14 inhibitor, and its role in TBI cannot be confirmed in our study, which also remains to be further elucidated.

The m6A methylation of mRNA after TBI has not been studied. The animal experiment results showed that the m6A methylation level changed significantly after TBI in rat cortex. These results in a rat TBI model might have meaningful correspondence to m6A related epigenetic changes in human studies [57]. Though we found that functional FTO is necessary to repair neurological damage caused by TBI, one limitation to our study was that it is not definitely clear the downstream targets of FTO. Future research could perform MeRIP-Seq on TBI + FB23–2 and TBI + DMSO rats to find out the direct target genes of FTO in TBI. Notably, FTO has been been found to be involved in leukemia, DNA damage repair, and heat stress [58].

## Conclusion

Our study showed that the *METTL14* and *FTO* were down-regulated after TBI in rat cortex. Through MeRIP-Seq, we identified the m6A methylation levels were significantly changed in 1580 mRNA. The following joint analysis of m6A peaks and mRNA expression revealed that there were 175 mRNA significantly changed after TBI. These genes may be the key genes to interfere in the epigenetic regulation of TBI. Moreover, we found that FTO played an important role in the maintenance of neurological function in TBI rats and could be a intervention target for alleviating damage caused by TBI.

## Supplementary information

**Additional file 1: Table S1.** PCR primers used in this study

**Additional file 2: Table S2.** The detailed information of raw data

**Additional file 3: Table S3.** Statistical analysis of reads mapped in reference genome

**Additional file 4: Table S4.** The m6A peak number on each gene

**Additional file 5: Table S5.** The detailed information of significantly changed m6A peaks

**Additional file 6: Table S6.** The detailed information of significantly changed mRNA

**Additional file 7: Table S7.** The detailed information of conjoint analysis between mRNA expression and m6A methylation

**Additional file 8: Table S8.** The raw data of mNSS tests

**Additional file 9: Table S9.** The raw data of MWM tests

## Data Availability

All of the datasets in this study can be obtained by reasonable request to the corresponding authors.

## Abbreviations

CCI: Controlled cortical injury
m6A: N6-methyladenosine
MeRIP-Seq: Methylated RNA immunoprecipitation sequencing
qRT-PCR: Quantitative reverse transcription polymerase chain reaction
TBI: Traumatic brain injury
