# Supplementary material for: Epitranscriptomic profiling of N6-methyladenosine-related RNA methylation in rat cerebral cortex following traumatic brain injury
Source: Mol Brain. 2020 Jan 28;13:11. doi: 10.1186/s13041-020-0554-0 (PMC6986156; doi:10.1186/s13041-020-0554-0)
Supplement: Supplementary file 1 — Additional file 1: Table S1. PCR primers used in this study [file 13041_2020_554_MOESM1_ESM.docx]

**Table S1.** Primers used in this study

| **Gene** | **Primer type** | **Primer Sequence （5’-3’）** |
| --- | --- | --- |
| *ACTB* | Forward | GGGAAATCGTGCGTGACATT |
| *ACTB* | Reward | GCGGCAGTGGCCATCTC |
| *ALKBH5* | Forward | ACGGCCTCAGGACATCAAAG |
| *ALKBH5* | Reward | AAGCATAGCTGGGTGGCAAT |
| *FTO* | Forward | AGGTCGAGTTTGAGTGGCTG |
| *FTO* | Reward | TTCACGAAGCACGGCATTTG |
| *METTL3* | Forward | ATGTGCAGCCCAACTGGATT |
| *METTL3* | Reward | CTGTGCTTAAACCGGGCAAC |
| *METTL14* | Forward | GCAGAAACCTACGCGTCCTA |
| *METTL14* | Reward | CACCACGGTCAGACTTGGAT |
| *WTAP* | Forward | CTCGCCTCGTCTCTTCTGG |
| *WTAP* | Reward | CATCTTGTACCCCGAGACGC |
| *VIRMA* | Forward | AAGGCCTCACCCTTGGAAAC |
| *VIRMA* | Reward | AAACAGTACTGCTCGGGGAC |
